# 

**Published:** 1919-06-07

**Authors:** 


### Reconstruction in Relation to Public Health.

An important Conference will be held in London from June 25 to June 28, under the auspices of the Royal Institute of Public Health, on Problems of Reconstruction in Relation to Public Health. The inaugural meeting on the 25th will be held in the Egyptian Hall of the Mansion House, and on the following three days the sessions of the Conference will be held in the Council Chamber. The matters for consideration fall under the following heads: (1) The Work of the Ministry of Health. (2) The Prevention and Arrest of Venereal Disease. (3) Housing in Relation to National Health. (4) Maternity and Child Welfare. (5) The Tuberculosis Problem Under After-war Conditions.

### MATRONS' AND SISTERS' APPOINTMENTS.

Bethnal Green Schools' Infirmary, Leytonstone.—Miss Florence Susan Cooper has been appointed sister. She was trained at Willesden Infirmary, has been staff nurse at Fulham Military Hospital and at the Manor War Hospital, Epsom, and sister at Barnet Infirmary. Miss Cooper holds the certificate of the Central Midwives Board.

Groesynyd Hospital, Conway.—Miss F. A. Young has been appointed sister. She was trained at Wandsworth Infirmary, and has been sister at the Royal National Sanatorium, Ventnor, Isle of Wight.

Beckett Hospital, Barnsley.—Miss M. R. Parsons has been appointed sister. She was trained at the Royal Hampshire County Hospital, Winchester, where she was later sister and assistant home sister. She has also served as sister under the French Flag Nursing Corps in France.

Miners' and Women's Hospital, Redruth.—Miss J. S. Lee has been appointed sister. She was trained at the Royal Infirmary, Liverpool, and Sea Croft, Leeds. She was sister of the London Fever Hospital, and has served three years in the T.F.N.S.

### THE COLLEGE OF NURSING (Limited by Guarantee).

### Sister-Tutor Studentship.

### EXAMINATION PAPER AND QUESTIONS, 1918.

INTENDING candidates entering for the examination required for sister-tutor studentships will be anxious and expectant as to the nature of the questions likely to be put before them. We have before us the syllabus of questions given at the last examination. These questions cover a wide range of subjects, and a knowledge of general information will prove a good asset to the candidate. Questions relative to medical and nursing subjects are well within the scope of ordinary knowledge and intelligence. The real test lies in the eighteen questions on subjects ranging over a wide area, ten of which are required to be answered. A little mental gymnastics will be needed to bring the mind from geography to history, literature to the poets, even an acquaintance with urban affairs may be useful. Here are a few examples of the subjects for a short essay:

1. The nursing profession as an asset to the State.
2. That discovery, invention, or other feature, which in your estimation has caused the greatest advance in nursing, or in treatment within recent years.
3. The relation of the nurse to the patient and to the doctor.

The subjects are mild in comparison with the questions on general knowledge, which include commercial points on exports and imports, and values relating thereto in various countries, and the names of certain personages making history, and a founder of Sunday schools. Then, again, with what events were Florence Nightingale and Robespierre associated? How much wall-paper 20 inches wide and 12 yards to the piece is required to paper a room (dimensions not given)?

To quote ten lines of your favourite poet, and give his name. To name the seas the Suez and the Panama Canals connect, and the commercial importance of the Dardanelles. Who are the authors of "Kim," "The Compleat Angler," "The Three Musketeers," and various well-known household books? Statistics of the mortality-rate in small hospitals, and how the history of nations is affected by the situation of the basins of certain rivers. Name approximate date of three American Presidents, and on how to engage a housemaid, in the most brief manner. The classification of the expenditure of a small hospital, as compared with a middle-class household, and why special countries provide topics of interest at the present time.

These extracts should cause a profundity of thinking on the part of those intending to enter for the scholarship examinations, but they need not cause any sense of the impossible or fear. Candidates this year are more fortunate than those entering last year, for they have here an intimation of what to expect, and how to attain victory.

### LONDON CENTRE.

(Hon. Secretary, Miss BIGGAR, St. Thomas's Hospital.)

WILL the members who have received their membership cards for the year April 1, 1919, to March 31, 1920, and have not already paid their subscription, kindly forward it to Miss Sordy, R.R.C., Queen Mary's Hospital for the East End, Stratford, E. 15? The subscription for the year April 1, 1919 to March 31, 1920, is 5s., and was payable in April of this year. At the members' general annual meeting it was decided that those who joined on or after January 1, 1919 and paid 5s. should only pay 2s. 6d. for this year; that those who paid 2s. 6d. on or after January 1, 1919, should pay the annual subscription, 5s.

We wish to draw attention to the fact that all arrangements for meetings, excursions, etc., will only be published in the nursing Press, and not given notice of by individual intimation. Notice of the June excursion will be published in the issue of the 13th inst.

It is hoped that many members of the London Centre's Club will be able to attend the fourth ordinary general meeting of the College of Nursing, Limited, which will be held on Wednesday, June 18, at 3 P.M., at the Victoria University, Manchester.

### NORTHUMBERLAND AND DURHAM CENTRE.

(Hon. Secretary: Miss F. JONES, Royal Victoria Infirmary, Newcastle.)

A PLEASANT social meeting was held on Wednesday, May 28, in the Royal Victoria Infirmary, Newcastle-on-Tyne. There was a good attendance, and some discussion concerning the Registration Bills now before Parliament. Music and singing followed, and was greatly appreciated. The next meeting will be held in the Royal Infirmary, Sunderland, on Saturday, June 28, at 3.30 P.M. All members are welcome.

### BIRMINGHAM CENTRE.

(Hon. Secretary, Mrs WILFRID CLEGG, 84 Hagley Road.)

THE annual meeting was held at the General Hospital on Saturday last. Miss Musson, R.R.C. (Matron of the General Hospital and Local Representative of the College), was in the chair. The annual report was read by the Hon. Secretary, Miss Colburn. Miss Musson was re-elected as the Local Representative, and Mrs. Boeddicker as Hon. Treasurer. Mrs. Wilfrid Clegg was elected Hon. Secretary in the place of Miss Colburn, retiring. The six members of the Executive Committee were then re-elected by ballot. Afterwards Miss Musson spoke on the work of the College of Nursing since its foundation, and its schemes for the future welfare of the nursing profession. She also drew attention to the serious questions connected with the two Bills for the Registration of Nurses now before Parliament, and to the urgent need for amendment in certain clauses in the Central Committee's Bill if it is to become law. The meeting ended with a vote of thanks to Miss Musson for her invaluable services to the Local Centre and to the nursing profession in general during the past year.

### LINCOLN CENTRE.

(Local Representative, Miss SHEPPARD, County Hospital, Lincoln.)

AT the Annual Meeting of this Centre, held on May 24, Miss Sheppard was unanimously re-elected Local Representative and Miss Kennedy Hon. Secretary. Miss Hobday (Assistant Matron Lincoln County Hospital) was elected Hon. Treasurer. The lectures held in this Centre are proving a great source of interest and increasingly well attended. Lieut.-Colonel Brook, R.A.M.C. (T.), gave a continuation of his lecture on "The Rôle of Psychology in Nursing," which was much appreciated, as was also a later lecture by Major Lowndes Yates, R.A.M.C. (T.), on "Advances in Nursing During the War."

### DERBY CENTRE.

(Hon. Secretary: Miss HAINES, Children's Hospital, Derby.)

THE members' meeting, formerly announced to be held on June 19, will now take place on June 20, at 5 P.M., at the Royal Infirmary, Derby. Miss Cowlin, Organising Secretary, will speak.

---

MAJOR T. VILLIERS CROSBY, R.A.M.C. (T.), M.D. London, senior honorary assistant physician on the staff of the Leicester Royal Infirmary, has been elected to the post of honorary physician.

